# Drug prescribing during pregnancy in a central region of Italy, 2008-2012

**DOI:** 10.1186/s12889-018-5545-z

**Published:** 2018-05-15

**Authors:** Martina Ventura, Alice Maraschini, Paola D’Aloja, Ursula Kirchmayer, Ilaria Lega, Marina Davoli, Serena Donati

**Affiliations:** 1 Department of Epidemiology, Latium Regional Health Servigce, Via Cristoforo Colombo, 112, 00147 Rome, Italy; 2 National Centre for Diseases Prevention and Health Promotion, Rome, Italy

**Keywords:** Inappropriate prescribing, Teratogens, Pregnancy, Health information system, Italy

## Abstract

**Background:**

Drug consumption during pregnancy is a matter of concern, especially regarding drugs known or suspected to be teratogens. Little is known about drug use in pregnant women in Italy. The present study is aimed at examining the prevalence, and to detect potential inappropriateness of drug prescribing among pregnant women in Latium, a region of central Italy.

**Methods:**

This retrospective study was conducted on a cohort of women aged 18-45 years who delivered between 2008 and 2012 in public hospitals. Women were enrolled through the Regional Birth Register. After linking the regional Health Information Systems and the Regional Drug Claims Register, women’s clinical data and prescribed medications were analyzed. Italian Medicine Agency (AIFA) and US Food and Drug Administration (FDA) evidence were used to investigate inappropriate prescribing and teratogenic risk.

**Results:**

Excluding vitamins and minerals, 80.6% (*n* = 153,079) of the women were prescribed at least one drug during pregnancy, with an average of 4.6 medications per pregnancy. Drugs for blood and hematopoietic organs were the most commonly prescribed (53.0%,), followed by anti-infectives for systemic use (50.7%). Among the inappropriate prescriptions, progestogen supplementation was given in 20.1% of pregnancies; teratogen drugs were prescribed in 0.8%, mostly angiotensin co-enzyme inhibitors (ACEIs) and angiotensin receptor blockers (ARBs) (0.3%).

**Conclusions:**

In Latium, drugs are widely used in pregnancy. Prescriptions of inappropriate drugs are observed in more than a fifth of pregnancies, and teratogens are still used, despite their known risk. Continuous updates of information provided to practitioners and an increased availability of information to women might reduce inappropriate prescribing.

## Background

Women may need to take drugs during pregnancy for pre-existing health problems (e.g. diabetes), conditions occurring during pregnancy (e.g. infections) or for pregnancy-related complications (e.g. gestational hypertension). However, information about the efficacy and safety of drugs in pregnancy is very limited because pregnant women are hardly ever included in drug clinical trials, mainly for ethical reasons [1]. Medication safety information in pregnancy is therefore obtained through case reports, observational studies and animal studies [2, 3], all of which have limitations, making the risk assessment of drug use during pregnancy difficult**.** All drugs should therefore be prescribed by assessing risks of medication against benefits of treatment on mother and fetus. It has been documented that congenital abnormalities caused by human teratogenic drugs account for less than 1% of total congenital abnormalities [4]. To evaluate the teratogenic or fetotoxic risks associated with drugs, different classifications systems based on data from human and animal studies have been developed. The US Food and Drug Administration (FDA) introduced the most well known classification in 1979 [5]. Medical associations have developed guidelines for the use of certain drugs during pregnancy including specific conditions or medication types [6, 7]. The EU-funded Seventh Framework Programme EUROmediCAT has developed a system to identify post market medications at the earliest possible stage that could increase teratogenicity. The program focused on risk assessment of congenital anomalies related to antiepileptics, insulin analogues, anti-asthmatics and antidepressant (SSRIs) and set out clinical recommendations [8]. In Italy, in 2005 the Ministry of Health appointed a panel of experts to release a clinical guideline on potential risks of drugs taken by mothers before and during pregnancy [9]. However, prescribing patterns continue to change over time as well as maternal characteristics such as age, pre-pregnancy BMI and clinical features. Therefore, prescribing contraindicated drugs to pregnant women is of great concern among clinicians and other obstetric health professionals. Intercountry comparability between studies on medication use in pregnancy is difficult due to dissimilarities in study design and methodology. A systematic review of recent literature on prescription drug use in countries with advanced health systems estimated that the number of pregnant women with at least one prescription, excluding vitamins and minerals, ranged from 44 to 93% [10]. A cross-sectional, multinational web-based study on the drug consumption in pregnant women showed that approximately 8 out of 10 women reported the use of at least one medication, either prescribed or over the counter, during the course of their pregnancy [11]. Studies providing additional information and updates on the use and appropriateness of medical drug consumption during pregnancy are of pivotal importance for women who are or are planning to become pregnant, for health care providers and for the society. So far, a limited number of studies has examined the use of prescription drugs among pregnant women in Italy [12–15]. The objective of this population-based study is the analysis of drug prescribing among pregnant women in Latium with specific regard to inappropriate and potentially or known teratogenic drugs.

## Methods

A cross-sectional population study was conducted using data available in the regional Health Information Systems, specifically the Regional Birth Register (CeDAP), the Hospital Information System (HIS) and the Regional Drug Claims Register (PHARM). Data from different information systems were combined using a deterministic record-linkage procedure based on anonymous identification codes, which is a procedure in line with privacy legislation.

All women residing in Latium aged 18-45 years, who delivered in the regional birth units from the 1st of January 2008 to the 31st of December 2012, were enrolled through CeDAP. Socio-demographic characteristics and clinical information related to pregnancy and to previous hospitalizations were collected from HIS. Drug prescriptions were retrieved from PHARM. This Register contains data on all drugs, reimbursable by the National Health Service, dispensed to residents in public and private pharmacies belonging to the regional health authorities. These drugs account for the majority of drugs used for acute or chronic conditions. Drugs administered during hospital stays are not registered. Data collection included information on the active agents and the exact quantification of the dispensed drug during pregnancy. We used the World Health Organization (WHO) Anatomical Therapeutic Chemical Classification System (ATC coding) to classify drugs, considering large anatomical groups, chemical groups (ATC fourth level) and single active agents (ATC fifth level) [16]. The gestational age at birth in completed weeks was retrieved by CeDAP, where it is routinely collected on the basis of the last menstrual period. In line with a study performed by Pisa et al., pregnancy trimesters were defined to assign etiologically relevant “time windows” of exposure to medication at the exact gestational age [17]. For each trimester, start and end dates were calculated backwards from the date of delivery and inferring the total pregnancy duration in days from the gestational age at birth.

Among women who gave birth more than once during the study-period, only information on medicines prescribed during the first pregnancy were recorded. Moreover, drugs prescribed only once during the first two months of pregnancy were excluded from the analysis, to avoid taking into account medications prescribed when the woman was not yet aware of being pregnant, and thus better assess the appropriateness of prescriptions in an ascertained pregnancy.

The main analysis focused on the drug prescription patterns in pregnancy. Analysis of drug prevalence was conducted both over the entire pregnancy and by trimester. Potential teratogens or potentially inappropriate drugs were assessed according to FDA and Italian Medicines Agency (AIFA) warnings by trimesters [5, 9]. The AIFA guide on drugs in pregnancy was considered as a reference document for Italian prescribers. A comparative analysis of the prescriptive behaviours before and during pregnancy was carried out for specific ATC groups.

Two logistic regression models were used: the first to model binary outcomes of potential inappropriate and teratogenic prescriptions, and the second to focus on inappropriate progestogen prescriptions. Model results were presented as odds ratios (OR) and their corresponding 95% confidence intervals (CIs). The independent variables included in the models were mother’s socio-demographic characteristics (age, nationality and education), parity, multiple pregnancy, previous miscarriages, comorbidities identified through HIS during the hospitalization for delivery and all hospital admissions in the previous two years. Comorbidities were first selected on the basis of a priori knowledge of clinical characteristics associated with outcomes and then grouped in a summary measure of severity.

The present study was funded by the Latium Region Pharmacovigilance call 2011 and was authorised by the ethics committee of the National Institute of Health.

## Results

During the five-year study period we identified 189,923 deliveries, 61.2% of women were primiparous and 92.7% of pregnancies ended at term. Slightly less than 40% of the mothers were older than 34 years and 89.6% were Italian citizens. The majority of women had an education of more than eight years of school (71.9%) (Table 1).

**Table 1:** Women’s socio-demographic and clinical characteristics

		Number	Percent
Age at delivery (years)	<=24	13,483	7.1
	25-29	36,524	19.2
	30-34	67,665	35.6
	35-39	56,622	29.8
	> = 40	15,629	8.2
Nationality	Italian	170,161	89.6
	Other	19,762	10.4
Level of education^a^	None or Elementary school (<=5 years)	9777	5.1
	Middle school (8 years)	42,535	22.4
	High school (12-13 years)	97,271	51.2
	Bachelor degree (> 13 years)	39,309	20.7
Gestagional age	Preterm (< 37 weeks)	13,289	7.0
	Full-term (37 e 42 weeks)	176,103	92.7
	Post-term (> 42 weeks)	531	0.3
Parity^a^	0	116,144	61.1
	1	58,587	30.8
	≥2	15,175	8.0

^a^The percentages may not sum 100% due to missing data

Excluding vitamins and minerals, 80.6% (*n* = 153,079) of the women were prescribed at least one drug during pregnancy, with a prevalence of 53.3, 57.4 and 49.1% by trimester, respectively. The average of medication prescriptions was 4.6 during the overall pregnancy and in almost half of the population, more than three prescriptions were registered (data not shown). The most frequently prescribed agents were drugs for blood and hematopoietic organs (53.0%, *n* = 100,663), with folic acid prescribed in 37% and iron in 26.3% of women, followed by anti-infectives for systemic use (50.7%, *n* = 96,251). Among anti-infective prescriptions, erythromycin was prescribed in 27.6%, amoxicillin in 13.5%, penicillin in 10.2% and tetracycline in 0.2% (Table 2).

**Table 2:** Women with at least one prescription during pregnancy: prescribed drugs according to ATC classification

Drug groups	ATC	Overall pregnancy	
		N	%
Anatomical groups			
Alimentary Tract and Metabolism	A	22,800	12.00
Blood and Blood Forming Organs	B	100,663	53.00
Cardiovascular System	C	5855	3.08
Dermatologicals	D	719	0.38
Genito Urinary System and Sex Hormones	G	41,178	21.68
Systemic Hormonal Preparations, excl.Sex Hormones and Insulins	H	21,037	11.08
Antiinfectives for Systemic Use	J	96,251	50.68
Antineoplastic and Immunomodulating Agents	L	244	0.13
Muscolo-Skeletal System	M	6291	3.31
Nervous System	N	3091	1.63
Respiratory System	R	17,273	9.09
Sensory Organs	S	301	0.16
Chemical subgroups *(most prescribed)*			
Folic acid and derivatives	B03BB	70,116	36,9
Macrolides	J01FA	52,438	27,6
Iron bivalent, oral preparations	B03AA	47,175	24,8
Pregnen derivatives	G03DA	38,834	20,4
Penicillins with extended spectrum	J01CA	25,581	13,5
Heparin group	B01AB	15,473	8,1
Thyroid hormones	H03AA	14,002	7,4
Glucocorticoids - respiratory	R03BA	12,587	6,6
Glucocorticoids - systemic	H02AB	8215	4,3

Overall, during pregnancy 44,303 women (23.3%) received potentially inappropriate clinical prescriptions, and 1420 women (0.8%) prescriptions of known or potential teratogens.

Among the group of clinically inappropriate drugs, progestogens were the most frequently prescribed (20.1%) (Table 3), particularly during the first trimester of pregnancy (17.1%), followed by glucocorticoids for systemic use (2.4%), propionic acid derivates (1.3%) and anti-inflammatory and anti-rheumatic drugs (1.2%) (Table 3).

**Table 3:** Prevalence of potential inappropriate/teratogen prescriptions during pregnancy according to trimesters at risk and ATC groups

Potential inappropriate drugs	ATC	Trimester at risk	N^a^	%
Total			44,303	23.33
Progestogens	G03D	1-2	38,146	20.09
Glucocorticoids for systemic use	H02AB	1	4458	2.35
Propionic acid derivates (Ibuprofen, Naproxen, Ketoprofen)	M01AE01-03	1-3	2495	1.31
Anti-inflammatory and antirheumatic	M01AX	1-3	2203	1.16
Acetic acid derivatives and related substances (excluding Indomethacin)	M01AB (exc M01AB01)	1-3	1461	0.77
Oxicams	M01 AC	1-3	200	0.11
Bile acid sequestrants	C10AC	1-3	191	0.10
Coxib	M01AH	1-2	40	0.02
Antigonadotropins and similar agents	G03XA	1-2	8	0.00
Indomethacin	M01AB01	1-2	8	0.00
Ezetimibe	C10AX09	1-3	1	0.00
Nicotinic acid and its derivatives	C10AD	1-3	0	0.00
Known or potential teratogen drugs	ATC	Trimester at risk	N^a^	%
Total			1420	0.78
ACE-inhibitors, ARBs or combinations	C09	2-3	508	0.27
HMG-CoA reductase inhibitors (statins)	C10AA,C10B	1-3	297	0.16
Barbiturates and derivatives	N03AA	1-3	199	0.10
Fatty acid derivatives	N03AG	1-3	177	0.09
Benzodiazepine derivatives	N03AE	1-3	132	0.07
Tetracycline	J01AA	3	91	0.05
Imidazole and its derivatives (Thiamazole)	H03BB02	1	48	0.03
Triazole and its derivatives (Fluconazole)	J02AC01	1	16	0.01
Lithium	N05AN01	1-3	16	0.01
Vitamin K antagonists	B01AA	1-3	13	0.01
Anti-arrhythmic drugs class III (Amiodarone)	C01BD01	1-3	9	0.00
Retinoids for acne treatment	D10BA01	1	4	0.00
Penicillamine and similar agents	M01CC	1-3	3	0.00
Retinoids for psoriasis treatment	D05BB	1-3	2	0.00

^a^women with at least one prescription

Among drugs defined as potentially teratogenic the most frequently prescribed were ACE-inhibitors (ACEIs) and ARBs or combinations (0.27%), followed by HMG-CoA reductase inhibitors (0.16%) and barbiturates and derivatives (0.10%), fatty acid derivatives (0.09%) and benzodiazepine derivatives (0.07%) (Table 3).

In a supplementary analysis focusing on antihypertensive drugs, we distinguished between prevalent and incident use of ACEIs/ARBs. Among the 3967 incident users, 550 started antihypertensive treatment during pregnancy. Of the 1446 women who had been taking ACEIs/ARBs before conception, 59.5% (*n* = 861) suspended the therapy, 25.9% shifted to alternative appropriate antihypertensive medications and 14.6% (*n* = 211) continued treatment with the teratogenic drug during pregnancy (data not shown).

Focusing on antiepileptic prescriptions (*n* = 898 women), we found that the mostly prescribed agents were carbamazepine, phenobarbital, valproic acid, clonazepam and lamotrigine. Two hundred and thirty four women were prescribed more than one antiepileptic drug (polytherapy) before pregnancy. Among these, 37% (n = 86) changed polytherapy treatment to monotherapy. Of the 204 women using high doses of antiepileptics before pregnancy, 42% reduced the dose after conception (data not shown).

Three logistic regression models showed factors associated with potential inappropriate, teratogenic and progestogen drug prescriptions, for which there is no scientific evidence for a benefit (Table 4). Maternal age ≥ 35 years and presence of comorbidities increased the risk of all three outcomes. Multiple pregnancies and previous miscarriage augmented the risk of inappropriate drugs and, particularly progestogen prescriptions. On the contrary, multiparity and high educational level were significantly protective factors for all potential inappropriate and teratogenic prescriptions investigated. Italian women had a higher risk of receiving progestogen prescriptions than foreigners (Table 4).

**Table 4:** Factors associated with potential inappropriate, teratogen, progestogen drug prescriptions during pregnancy

		Inappropriate drug prescriptions^a^			Teratogen drug prescriptions			Progestogen drug prescriptions			
		%	OR adj	IC 95% (inf-sup)	%	OR adj	IC 95% (inf-sup)	%	OR adj	IC 95% (inf-sup)	
Socio-demographic variables											
Age classes	≤34	5.4	1		0.7	1		15.8	1		
	35-39	7.8	1.54	1.47-1.61	0.9	1.45	1.29-1.64	25.4	1.88	1.83	1.92
	≥40	11.4	2.23	2.08-2.38	1.2	1.99	1.68-2.35	33.5	2.68	2.58	2.79
Nationality	Other	6.4	1		0.8	1		12.9	1		
	Italian	6.5	0.95	0.89-1.01	0.8	0.97	0.81-1.15	20.9	1.52	1.46	1.59
Education	None/elementary school	7.7	1		1.1	1		20.3	1		
	Middle school	6.9	0.99	0.90-1.08	0.8	0.79	0.63-0.98	17.8	0.99	0.93	1.05
	High school	6.5	0.86	0.79-0.94	0.8	0.67	0.55-0.83	20.4	1.04	0.99	1.10
	Bachelor degree	5.9	0.72	0.65-0.79	0.7	0.57	0.45-0.72	21.8	0.97	0.92	1.02
Other variables											
Multiple pregnancies	No	6.3	1		0.8	1		19.5	1		
	Yes	21.1	3.75	3.37-4.17	0.9	1.03	0.71-1.50	50.9	3.91	3.65	4.19
Parity	Primiparous	6.7	1		0.8	1		21.9	1		
	Multiparous	6.2	0.77	0.74-0.81	0.8	0.85	0.76-0.95	17.3	0.63	0.61	0.64
Previous miscarriages (10 years before)	None	6.1	1		0.8	1		18.7	1		
	At least one	12.5	2.02	1.90-2.15	0.8	0.91	0.75-1.11	35.3	2.19	2.11	2.27
Comorbidities^b^											
Comorbidities	None	6.4	1		0.7	1		19.9	1		
	1	10.3	1.57	1.44-1.71	1.5	2.06	1.68-2.51	24.4	1.18	1.11	1.24
	≥2	16.1	2.63	2.06-3.36	4.5	6.15	4.13-9.15	26.4	1.28	1.07	1.54

^a^women who had only progestogen prescriptions were not considered

^b^anemia, coagulation defects, cardiovascular disorders, congenital heart defects, congenital malformations of circulatory system; cerebrovascular disorders nephritis, nephrotic syndrome, and nephrosis; collagen diseases, HIV seropositive/AIDS; thyroid diseases; diabetes, hypertension, chronic obstructive pulmonary disease, asthma, cystic fibrosis, chronic pneumonia

## Discussion

The estimated rate of drug prescriptions in pregnancy among women delivering in Latium from 2008 to 2012 was 81% excluding vitamin and minerals. To our knowledge the only two other population-based studies conducted in 2004 and in 2011 in two regions of northern Italy [13, 15] reported respectively 48,3 and 72,7% of antenatal prescriptions.

In developed countries the use of prescription medicines is widespread, but comparisons are difficult due to differences in methods and reporting of drug utilization. A systematic review published from 1989 to 2010 reported a wide variation in estimates of overall antenatal drug prescriptions, ranging from 27 to 93%. Among the most comparable European studies using administrative prescription databases, Northern Europe reported the lowest rates of prescriptions ranging from 44.2 to 57%. The highest rates were found in the Netherlands (69.2%), Germany (85.2%) and France (93%) [10].

In our study, the most frequently prescribed agents were drugs for blood and hematopoietic organs (53%) with folic acid prescribed in 37%. This proportion underestimates the real consumption because in Italy folic acid is available as an over the counter drug. A knowledge, attitude and practice survey investigating the use of folic acid and its appropriateness among a sample of 562 women who delivered in Latium region between 2013 and 2014 [18] reported 95% of women had taken folic acid during pregnancy. In line with the increase (13%) in antibiotic consumption registered in the Italian general population in the years 1999-2007, in our study 50% of the participating women took antibiotics during pregnancy [19]. This proportion is higher compared to other population-based studies in European settings ranging from 27 to 32% [20–22] and similar to a French study where 42% of pregnant women were exposed to antimicrobials [23]. The previous Italian studies reported respectively 37.2 and 24.8% of women prescribed with antibiotics during pregnancy [13, 15]. Given the growing problem of bacterial resistance, an increase in the use of antibiotics during pregnancy requires careful thought in terms of prescriptive appropriateness [24]. Therefore, it is urgent to understand the motivation for the high antimicrobial prescription rate in Latium. The most likely patients to be prescribed these drugs were women aged over 40 representing 8% of the total cohort, and those who had comorbidities during hospitalizations in the previous two years (data not shown).

The frequent prescribing of progestogens (21.2%) during the first and second trimester of pregnancy detected in Latium is worthy of attention. In 2009, the World Health Organization recommended not to prescribe progestogens for preventing miscarriages. A Cochrane review updated in 2013 [25], including respectively 14 RCTs (2158 women), concluded that progestogen is ineffective for the treatment of threatened abortion except in women with a history of three or more previous miscarriages. On the other hand, a more recent randomized trial in which progestogen had been administered during the first trimester of pregnancy did not result in a significant increase in the rate of live births even among women with a history of unexplained recurrent miscarriages [26]. The evidence that progestogen is ineffective in lowering the risk of miscarriages and the persistence of this inappropriate prescriptive habit in Italy bolsters the importance of clinical recommendations to avoid unnecessary medication overuse which increases public spending .

Seven studies included in the Canadian systematic review reported the use of drugs recognized as having potential risks for fetal harm in pregnancy, as classified by the FDA, ranging from 0,9 to 4,6% [10]. In the present study, the percentage of pregnancies exposed to known or potential teratogens was 0.7%.

Antihypertensive drugs such as ACE inhibitors and ARBs are extremely effective in lowering blood pressure and offer significant benefits in proteinuric diseases. Their use during the second and third trimesters is contraindicated by FDA because their in utero effect is associated with fetal renal dysplasia, anuria and kidney failure, and even fetal death [27–29]. In our study ACE inhibitors and ARBs were prescribed in 0.3% of the Latium cohort, showing rather similar prescribing patterns as compared to those reported in Lombardia between 2009 and 2010 [14]. Hence, prenatal counseling in women with chronic hypertension is an important component of their care. Women should be advised on the importance of birth control while on these drugs and should promptly suspend their intake in case of pregnancy, switching to a safe antihypertensive therapy in case dietary restrictions are ineffective in controlling blood pressure.

Among drugs defined as known or potential teratogens, the ATC group of anti-epileptics deserves different considerations because interruption of the treatment could pose even greater risk for both the mother and the fetus. Recommended solutions include switching from combination to monotherapy and prescribing the minimum effective dosage. In our study, 37% of women switched from polytherapy to monotherapy and 42% reduced their dose, suggesting that clinicians were aware of the appropriate clinical practice.

Multivariate data analysis (Table 4) assessed a lower risk of receiving inappropriate and teratogenic prescriptions during pregnancy among highly educated and multiparous mothers probably due to their greater “empowerment” compared to lesser educated women and first-time mothers. Contrarily, one or more previous comorbidities and maternal age ≥ 35 years were significantly associated with a higher risk of potential inappropriate and teratogenic prescriptions. As more women delay childbearing in Italy, where the proportion of delivering women ≥35 years rose from 9% in 1981 to 35% in 2014, advanced maternal age is a challenge for the health system.

The logistic regression investigating risk factors for clinically inappropriate prescribing gave evidence that multiple pregnancies and a history of previous miscarriage are associated with an almost four times higher risk of inappropriate and teratogenic prescriptions compared to singleton pregnancies, and to a double risk compared to women without previous miscarriages. On the contrary, multiparous women were found to be at lower risk of inappropriate, teratogenic and progestogen prescriptions. We discussed these findings with a panel of gynecologists, general practitioners, and midwives, and they suggested that multiparous women may be more experienced and better informed compared to nulliparae and therefore, less likely to overuse medicalization that characterizes pregnancy care in Italy.

There are some limitations to be mentioned: between-country comparison may be biased by different drug reimbursement policies, i.e. some drugs that are not refunded in Italy might be refunded in other countries and vice versa. The present results refer to one Italian region and may not be representative of other parts of Italy.

Furthermore, using administrative data, drug use may be prone to imprecisions in either direction, over- and under-estimation: our data do not include *over-the-counter* drugs or drugs which are not refunded by the health care system. On the other hand, drug use could be overestimated, in case the drug is claimed in the pharmacy but not actually taken by the woman. Previous investigations have shown a good capacity of the Italian health information systems to capture medications used chronically, whereas drugs used sporadically may be underestimated or not correctly allocated in time (e.g. in Italy, antibiotics are usually stocked at home and taken as needed, which means that the user and the time of use may not match with the purchaser and the time of the drug claims registered in our administrative data). This observation is in line with results reported in Canada [30], the Netherlands [31] and the US [32]. A recent Italian study comparing administrative data with maternal self-reports on drug use in pregnancy found a high agreement for medications used for chronic conditions. The authors also investigated the quality of information retrieved from administrative data on gestational age and found birth certificates to be a reliable source of information on the timing of pregnancy [17]. Moreover, misclassification associated to socio-economic differences can be ruled out as shown in a previous study which gave evidence for equal access in our health care system [33]. Unfortunately our database does not provide information on indications for prescribing, and consequently we were not able to investigate the use of these drugs more in depth. Finally, the present study could not include pregnancies ending in spontaneous and therapeutic abortions, because they are not retrievable in the database. This limitation introduces a likely underestimation of drug prescribing with a potential for fetal harm. On the other hand, the population based approach, the record-linkage of administrative databases and the large cohort enrolled are strong points of the study, allowing robust data analysis reinforced by the sensitivity analysis for determining the prescription patterns.

Previously, four studies on this topic were performed in Italy. However, one referred to a population survey including a sample of Italian women and contained only a few questions on drug use [12], and a second one was an investigation focusing on antihypertensive drug use in pregnancy [14]. The two population-based studies using administrative data were conducted in northern Italy [13, 15] and may not be representative of other Italian contexts and geographical areas and this was a motivation to conduct the study in Latium. It has been previously shown that between Italian geographical areas and regions there is a considerable variability in the prevalence of drug consumption during pregnancy. For example, the consumption of folic acid during the periconceptional period varied between 0.0 and 40.0% and between 7.1 and 39.5% according to different geographical areas in two wide multi-centric population based surveys [34, 35]. Therefore, this is the first population-based study in a region located in central Italy, evaluating prescription drugs in detail during pregnancy in recent years.

In Latium, drugs are widely used in pregnancy and the present study highlights the necessity of specific interventions in three distinct areas; first, the periodic collection of data regarding drug prescribing during pregnancy; second, the continuous update of information provided to practitioners (specifically general practitioners and gynecologists); and third, the increased availability of information to women and the public. In view of the evident differences based on the womens’ educational level, reducing inequities in access to information and drug prescribing is a matter of priority.

This project can be considered a pilot study able to detect and investigate the critical aspects of drug prescribing in pregnancy in a large Italian cohort of pregnant women. The adopted method could be periodically replicated in different regions of the country with the objective of monitoring prescription patterns in pregnancy, and make recommendations to prevent prescriptions of potential teratogens and clinically inappropriate drugs. Our study is in line with the objectives of EUROmediCAT project, which plans to build a European system that allows evaluating the safety of drug use during pregnancy.

## Conclusions

Drug prescriptions during pregnancy in Latium are very common. Consumption of potential teratogens and clinically inappropriate drugs was low but not absent. Continuous update of information provided to practitioners, and an increased availability of information to women and the public may help to further reduce the risk of inappropriate prescribing during pregnancy.

## Abbreviations

ACEI: Angiotensin Co-Enzyme Inhibitor
AIFA: Agenzia Italiana del Farmaco
ARB: Angiotensin Receptor Blocker
ATC: Anatomical Therapeutic Chemical classification
BMI: Body Mass Index
CeDAP: Certificato di Assistenza al Parto
CI: Confidence Interval
EU: European Union
FDA: Food and Drug Administration
HIS: Hospital Information System
OR: Odds Ratio
PHARM: Regional Drug Claims Register
RCT: Randomized Controlled Trial
SSRI: Selective serotonin reuptake inhibitor
WHO: World Health Organization
